# Development and preliminary evaluation of a novel preoperative index for quantitative analysis of photoreceptor loss in full-thickness macular holes

**DOI:** 10.1007/s00417-024-06654-z

**Published:** 2024-10-26

**Authors:** Alberto Quarta, Andrea Govetto, Annamaria Porreca, Lisa Toto, Marta Di Nicola, Maria Ludovica Ruggeri, Matteo Gironi, Mario Nubile, Luca Agnifili, Mario R. Romano, Rodolfo Mastropasqua

**Affiliations:** 1 Department of Medicine and Science of Ageing, Ophthalmology Clinic, University “G. d’Annunzio”, National Center of High Technology in Ophthalmology Via dei Vestini, 66100 Chieti-Pescara, Chieti Italy; 2 Ophthalmology Department, Vitreoretinal Division, Ospedale Circolo e Fondazione Macchi, Varese, Italy; 3 Laboratory of Biostatistics, Department of Medical, Oral and Biotechnological Sciences, University “G. d’Annunzio” Chieti-Pescara, via dei Vestini 31, 66100 Chieti, Italy; 4 Ophthalmology Department, Humanitas Gavazzeni Hospital, Humanitas University, Bergamo, Italy; 5 Department of Neurosciences, Imaging and Clinical Sciences, University “G. d’Annunzio” Chieti-Pescara, Chieti, Italy

**Keywords:** Full-thickness macular hole, Photoreceptors, Macular hole borders, PIIN

## Abstract

**Purpose:**

To identify novel quantitative parameters for evaluating photoreceptor loss in full-thickness macular holes (FTMH), exploring their potential clinical impact on postoperative functional and anatomical recovery.

**Methods:**

This pilot study enrolled 38 eyes from 38 patients diagnosed with FTMH. Preoperatively, eyes underwent analysis and were subsequently followed for six months post-surgery. Best-corrected visual acuity (BCVA) was recorded, and cross-sectional images of FTMH were obtained using B-scan optical coherence tomography (OCT) and en-face OCT. Quantitative assessment of ellipsoid zone (EZ) and external limiting membrane (ELM) integrity changes was conducted and correlated with postoperative anatomical and functional recovery. The photoreceptor Integrity Index (PIIN), calculated as the ratio of photoreceptor area to lumen hole area measured at customized segmentation, was correlated with the minimum and base diameters of the hole, positive change in BCVA, preoperative EZ defect (EZd), preoperative ELM defect (ELMd), and changes in EZ and ELM over the six-month follow-up period (∆-EZ and ∆-ELM). The main outcome measures focused on evaluating the effectiveness of PIIN in predicting postoperative anatomical and functional changes.

**Results:**

A higher PIIN correlated with a greater BCVA change over six months (p < 0.001). Univariate regression analysis using the PIIN as a predictor for positive change in BCVA (|∆-BCVA| [logMAR]) over time yielded significant results (p < 0.001). Additionally, the PIIN significantly correlated with EZd at baseline, ELM at baseline, and ELMd change over the six-month follow-up period.

**Conclusion:**

The PIIN shows promise as a tool for evaluating photoreceptor loss in macular holes and estimating postoperative functional and anatomical recovery.

**Key messages:**

*What is known*

- Previous studies have extensively used optical coherence tomography (OCT) to investigate various biomarkers for assessing patients with full-thickness macular hole (FTMH), without considering detailed MH ultrastructural features
- Existing indexes used to predict surgical outcomes for FTMH primarily depend on geometrical parameters and do not integrate detailed ultrastructural characteristics, such as cellular components.

*What is new*

- A novel concept introduces the quantitative measurement of residual photoreceptors located at the edge of FTMH.
- The Photoreceptor Integrity Index (PIIN) integrates different ultrastructural components of macular holes, aiming to become a valuable clinical tool to predict both anatomical and functional recovery outcomes following surgical intervention for FTMH.

## Introduction

Idiopathic full-thickness macular holes (FTMH) are often well characterized by optical coherence tomography (OCT) with either structural OCT B-scan and en-face OCT [1, 2]. The latter has gained importance in evaluating vitreomacular disorders, with well-described applications in assessing the structural characteristics of MHs [3, 4].

Nevertheless, reliable preoperative OCT metrics able to provide an accurate prediction of visual potential after surgery are still lacking [5]. Although modern surgical techniques ensure a high hole closure rate, visual recovery may be suboptimal [6–8].

Recently, Govetto et al. [9] described a new OCT qualitative metric by analyzing the border hole morphology, which they categorized into two subtypes: smooth and bumpy borders.

In their study, bumpy borders were associated with photoreceptor disruption, negatively influencing functional and anatomical recovery. Although the focus of the Govetto et al. study is on the number of photoreceptor cells lost, a deeper insight into the MH potential recovery after surgery could be done by focusing on residual photoreceptor cells on the hole hedge. Histopathological studies showed that photoreceptors at the hole edge are the most representative site of residual photoreceptor survival since they represent the furthest point from the choroid, responsible for 70-80% of the outer retina nutrients [10, 11].

In this study, we used en-face OCT to characterize and quantify photoreceptor remnants at the hedge of FTMHs. Moreover, we elaborated a novel prognostic index related to photoreceptor integrity to predict anatomical and functional restoration and tested its potential clinical significance.

## Materials and methods

### Design and population study

This pilot study was conducted with the approval of the Institutional Review Board of the University "G. d'Annunzio," Chieti-Pescara, Italy. All procedures used adhered to the tenets of the Declaration of Helsinki. Informed consent for the research was obtained from the patients after an explanation of the nature and possible consequences of the study. The study population comprised 38 consecutive patients diagnosed with FTMH who underwent surgery at the University Hospital of Chieti-Pescara Ophthalmology Clinic between January 2022 and January 2023.

All patients underwent a comprehensive ophthalmological examination, including measurement of best corrected visual acuity (BCVA) expressed in logarithm of the minimum angle of resolution (logMAR), intraocular pressure (IOP) measurement with Goldman applanation tonometry, fundus examination with indirect ophthalmoscopy after 1% tropicamide instillation, multimodal imaging with Optical Coherence Tomography (OCT) and Optical Coherence Tomography Angiography (OCTA) at baseline and follow-up visits. Axial length was measured using partial coherence laser interferometry (IOL Master 700; Carl Zeiss Meditec, Jena, Germany). The duration of symptoms was also recorded. Postoperative follow-up was conducted at six months. All imaging was performed by a single-trained ophthalmologist (AQ).

Inclusion criteria comprised the presence of an idiopathic FTMH treated with surgery within six weeks from the diagnosis and macular hole closure after a single surgery. Exclusion criteria included high myopia (defined as a refractive error equal to or more than -6 diopters and an axial length equal to or more than 26.6 mm), intraoperative and postoperative complications, secondary and atypical macular holes, any other vascular retinal disease, uveitis and glaucoma, history of intraocular surgery (except for uncomplicated phacoemulsification).

## Multimodal imaging

All patients underwent OCT scan with SD-OCT (Spectralis HRA + OCT; Heidelberg Engineering, Germany) and SS-OCTA/en-face scan with Plex Elite (Plex Elite 9000; Carl Zeiss Meditec, Inc).

OCT scans with Spectralis included single horizontal and vertical high-definition scans passing through the center of the macular hole and dense scans (97 scans x 60 µm). OCTA and en-face scans were performed with a 3 mm x 3 mm scanning pattern. Only scans with an acceptable signal strength index (SSI) > 8 were accepted for analysis.

All macular holes were classified according to the International Vitreomacular Traction Study Group classification (IVTS) [12].

## Quantitative variables analyzed

### B-Scan optical coherence tomography measures

Starting From Linear Scans centered at the macular hole, minimum hole diameter, basal diameter, ellipsoid zone defect (EZd), and external limiting membrane defect (ELMd) were measured by using Heidelberg caliper tool. EZd and ELMd were defined as the distance between EZ and ELM terminals on each side of the hole.

### EN Face optical coherence tomography acquisition

To assess residual photoreceptors at the MH edge an indirect en-face OCT biomarker was considered such as ELM presence at the highest retinal point along the 360° macular hole border.

**Fig. 1:**
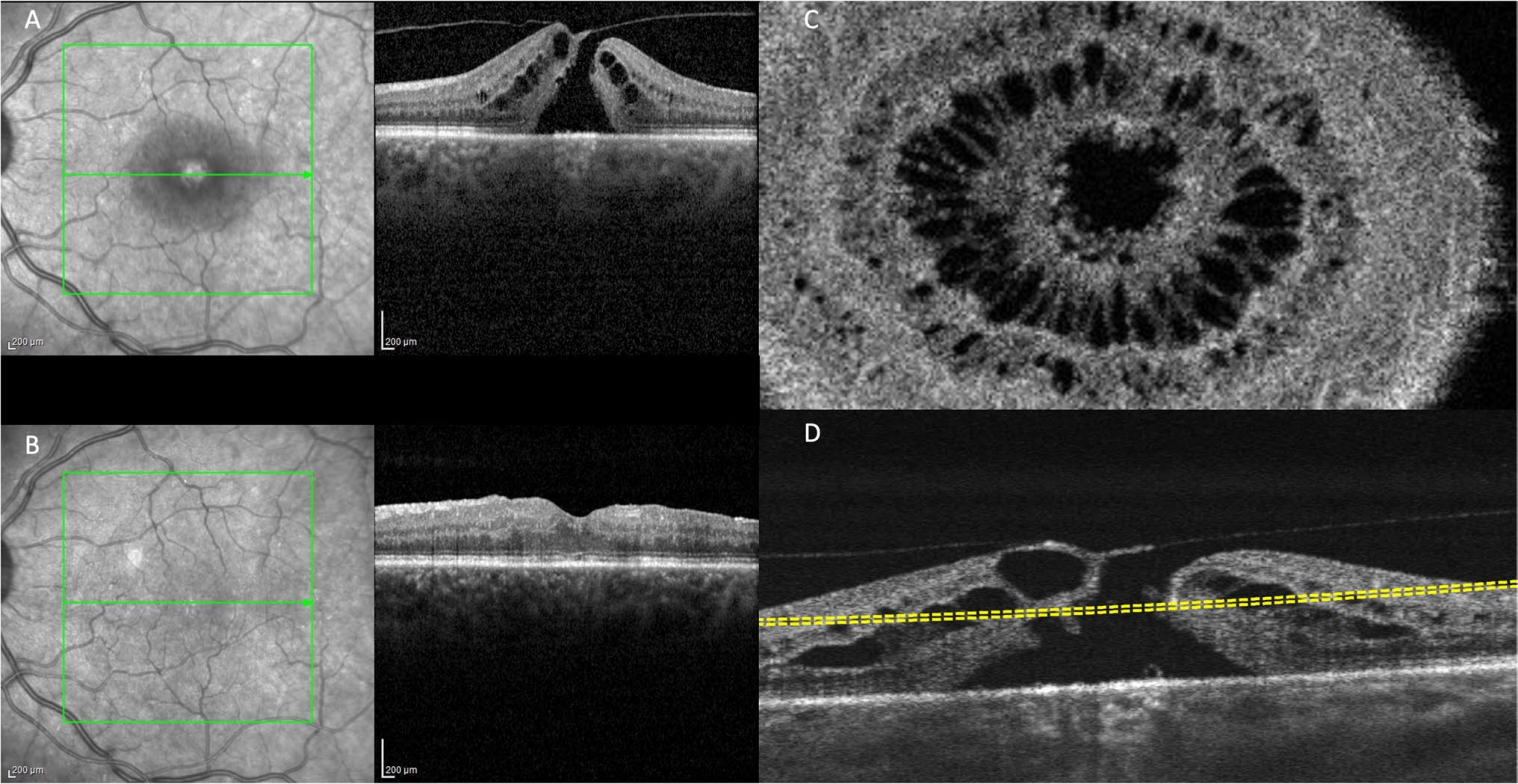
Representative optical coherence tomography [OCT] of a 65-year-old woman left eye with full thickness macular hole. [Upper left] B-scan OCT showing macular hole with irregular border before surgery (**A**) and after surgery at 6 months (**B**). En face 3 x 3 mm scan at the ELM end over the border, obtained by segmentation performed on lower right scan (**D**). [Upper left] Corresponding En-face OCT scan showing the slab set to visualize the photoreceptors at the hole hedge (**C**)

En-face structural 3 mm x 3 mm scans from OCTA (corresponding to 300 A-lines/B-scan and 300 B-scans acquired at 100kHz) were performed. Horizontal spectral-domain optical coherence tomography B-scan of the FTMH was manually segmented at the hole edge where the ELM was visible. For this layer, to perform a linear segmentation, we take the 20-µm thick choriocapillaris slab shifted to the mentioned point as presented in Figure 1.

### Photoreceptor integrity index

**Fig. 2:**
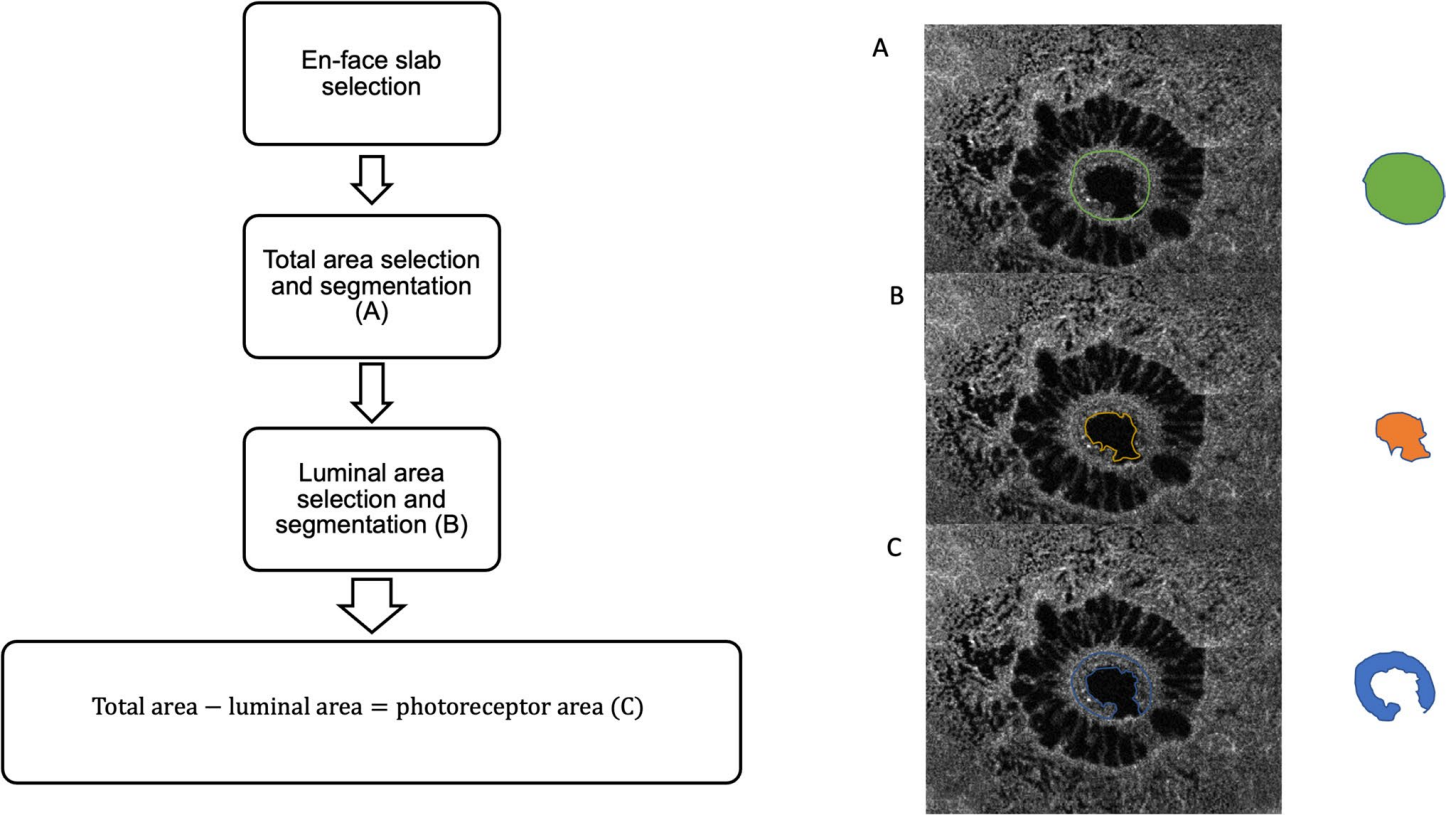
Representative En-face OCT scans with the proposed algorithm performed. Scan on the right shows boundary of the hole area (**A**), on the center boundary of lumen of the hole (**B**) and on the right the difference between the two (**C**), representing photoreceptors remnant area

To quantify the photoreceptor at the hole edge, we elaborated a novel measure standardizing it. Using the Plex Elite caliper-free tool, on the corresponding B-scan of the en-face acquisition, we selected the temporal and nasal hole edges through the slice navigator tool to obtain an anatomical reference for the en-face area selection analysis (Figure 2). The key features indicative of photoreceptor integrity were obtained through a three-steps approach. First, the total area (TA) of the hole was accurately segmented in the selected slab, considering as reference a hyperreflective circumferential line which represents the hole edge where photoreceptors lodge (Figure 1A). This selection was adopted to exclude the retinal area with cystic spaces since the analysis was focused only on the hole edges. Second, we segmented the luminal area (LA) (Figure 2B), then we subtracted it from the TA to obtain the photoreceptor area (PRA), as illustrated in Figure 2C. All measurements were performed by two experienced ophthalmologists (AQ, LT). If no intergrader agreement could be obtained for anatomical reference, the final agreement was confirmed by consulting a senior investigator (RM), who addressed any issues regarding variability in identifying the precise boundaries of the photoreceptor layer, the difficulty in segmenting the luminal area due to variations in image quality, and the challenges associated with accurately defining the hyperreflective circumferential line representing the photoreceptor edges. Because the PRA is not a pure measure and is influenced by hole size, we elaborated a new index named the ''Photoreceptor Integrity Index'' (PIIN) as a measure for residual cells of the hole edge.

We obtained this index by the formula:

Normalizing Photoreceptor Remnants Area (mm^2^) and Luminal Area (mm^2^) by the min-max method [13]:

$$\mathrm{Normalized}\;\mathrm X\;\mathrm{Variable}=\frac{\mathrm X-\mathrm{MAX}\lbrack\mathrm X\rbrack}{\mathrm{MAX}\left(\mathrm X\right)-\mathrm{MIN}\lbrack\mathrm X\rbrack}$$

We obtained two new normalized variables, Normalized Photoreceptor Remnants Area (NORM-PRA) and Normalized Luminal Area (NORM-LA).

Thus, avoiding dividing for zero, the new PIIN is:

$$PIIN =\frac{\text{NORM}-\text{PRA }}{\text{NORM}-\text{LA}+1}$$

Because the NORM-PRA and NORM-LA vary from 0 to 1, our index is defined by non-zero total area, the 0 ≤ PIIN ≤ 1. The domain of this new index is between 0 and 1. Values close to zero mean poor photoreceptor survivor at the hole hedge, and values close to 1 mean more exposed photoreceptors survive.

### Surgical procedure

All patients underwent a 25-gauge pars plana microincision vitrectomy system after subtenon anaesthesia (Constellation; Alcon Laboratories, Inc) performed by a single expert vitreoretinal surgeon (RM). Combined phaco-vitrectomy and intraocular lens implantation (IOL) were performed in the case of phakic eyes. Traditional core vitrectomy with posterior vitreous detachment (when not present) was performed. Then, the ILM was stained for 1 minute with Membrane Blue Dual dye (DORC, Rotterdam, the Netherlands) with the microscope light turned off, and the internal limiting membrane was peeled starting from the temporal arcade. All cases were tamponated with 20% sulfur hexafluoride (SF6). Each patient followed face-down positioning for three days.

### Statistical analysis

Intergrader repeatability was assessed by computing the concordance correlation coefficient (CCC) and its 95% Confidence Interval (CI)**.** Descriptive statistics include median, q1 = first quartile, q3 = third quartile, minimum and maximum value. Categorical variables are summarized by absolute frequency (%). Normality was tested by using the Jarque-Bera test. The Wilcoxon rank sum test was used to compute differences in EZ (μm), ELM (μm) and BCVA (logMAR) from baseline (T0) to follow-up (T1). Spearman’s ρ correlation coefficient with listwise deletion was calculated to assess the relationship between the continuous parameters and PIIN. A univariate linear regression model assessed how the PIIN influenced the positive change of BCVA (logMAR) between the baseline and the follow-up. All the statistical tests were two-sided, with a significance level set at p ≤ 0.05. The R-squared expresses the model performance. Statistical calculations were performed using R software environment for statistical computing and graphics (version 4.1; http://www.r-project.org/).

## Results

### Study population

Out of the initial 45 cases of FTMH included in the study, 7 cases failed to close following the initial surgery and were subsequently excluded from the analysis. Of the 38 patients included in this analysis, 23 were females (60.5%), and 15 were males (39.5%). Median value age was 69.5 years (67.2;75.0). All eyes had a follow-up of six months. All patients were phakic, and a combined vitrectomy was performed in each case. No intra or postoperative complications occurred. The median duration of symptoms was 4.00 months (3.00-7.00).

### Functional analysis

**Fig. 3:**
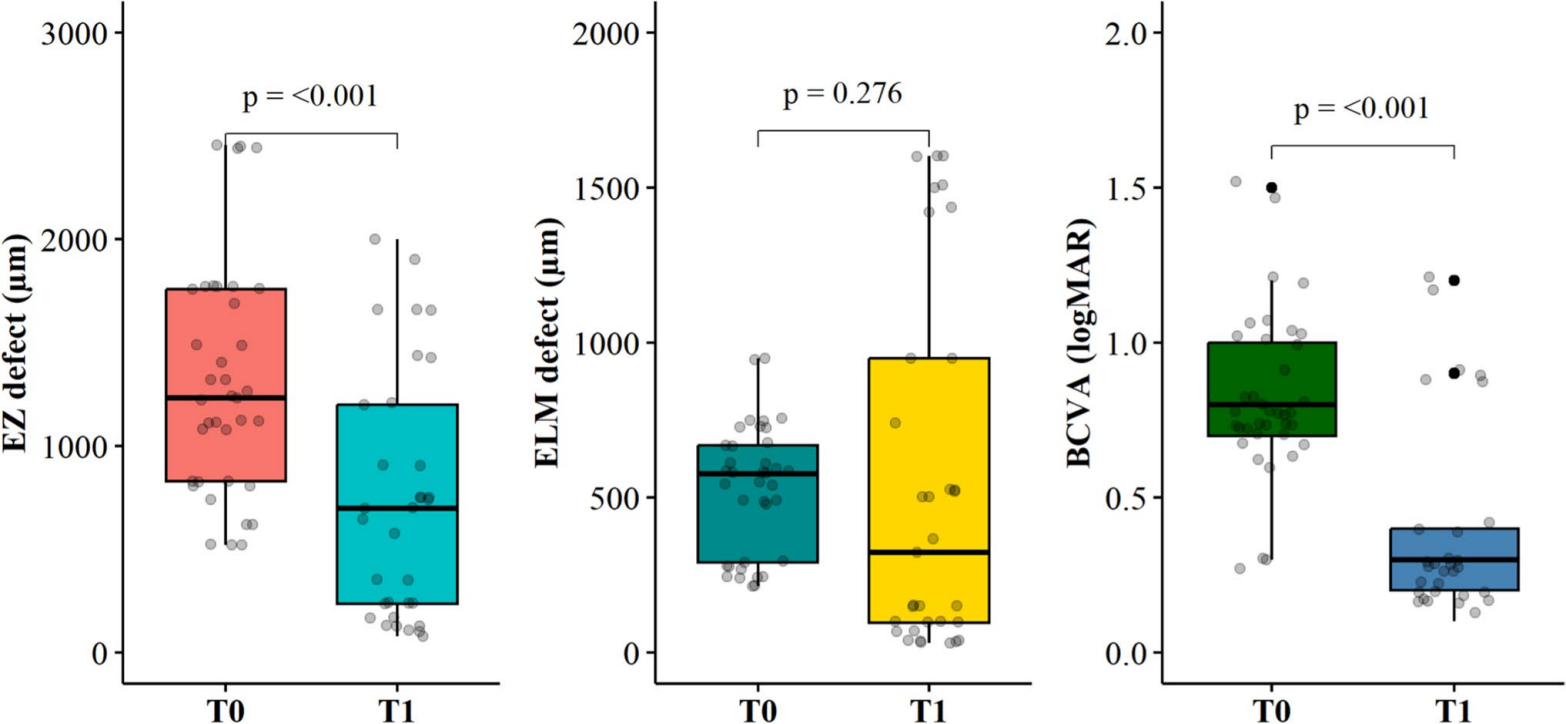
Box plots for EZ defect [μm], ELM defect [μm] and BCVA [logMAR] at Baseline [T0] and Follow-up [T1]. P-values derived from the Wilcoxon rank sum test

Significant improvement in BCVA from 0.80 (0.70-1.00) logMAR at baseline to 0.30 (0.20-0.40) logMAR at six months was registered (Figure 3). ∆-BCVA was 0.40 (0.30-0.50) logMAR.

### Optical coherence tomography b-scan analysis

According to IVTS, 25 were large full-thickness macular holes (66%), and 13 were medium holes (34%). Table 1 resumes baseline and post-operative OCT patients’ characteristics.

**Table 1:** Summary descriptive statistics of the baseline patients’ characteristics expressed as median, q1= first quartile, q3=third quartile, maximum value, and minimum values. For gender the variables and absolute frequency is reported

*N***=**38 (23 F, 15 M)	Median	q1	q3	Max	Min
Months of Symptomps	4.00	3.00	7.00	10.00	1.00
Age, years	69.50	67.00	75.00	82.00	56.00
Basal Diameter (µm)	860.00	652.00	1159.00	1475.00	322.00
Minimum Diameter (µm)	492.00	279.00	622.00	741.00	226.00
LA (mm^2^)	0.15	0.06	0.25	0.53	0.03
NORM-LA	0.24	0.05	0.44	1.00	0.00
PRA (mm^2^)	0.10	0.06	0.16	0.29	0.03
NORM-PRA	0.27	0.12	0.50	1.00	0.00
PIIN	0.17	0.04	0.32	0.66	0.00
TA (mm^2^)	0.30	0.12	0.42	0.76	0.06
BCVA (logMAR) at T0	0.80	0.70	1.00	1.50	0.30
|∆-BCVA| (logMAR)	0.40	0.30	0.50	0.80	0.00
EZ (µm) at T0	1234.00	830.00	1759.00	2456.00	520.00
|∆-EZ| (µm)	567.00	392.00	1061.00	2101.00	78.00
ELM (µm) at T0	578.00	291.00	670.00	948.00	214.00
|∆-ELM| (µm)	215.00	180.00	451.00	933.00	2.00

*LA* Luminal Area, *PRA* Photoreceptor Area, *TA* Total Area, *PIIN* Photoreceptor Integrity Index, *BCVA* Best Corrected Visual Acuity, *EZ* Ellipsoid Zone, *ELM* External Limiting Membrane. |∆|=change from follow-up (T1) to baseline (T0) in absolute value. *F* females, *M* males. *N* total number of patients

### EN-face optical coherence tomography of hole edge photoreceptors

**Fig. 4:**
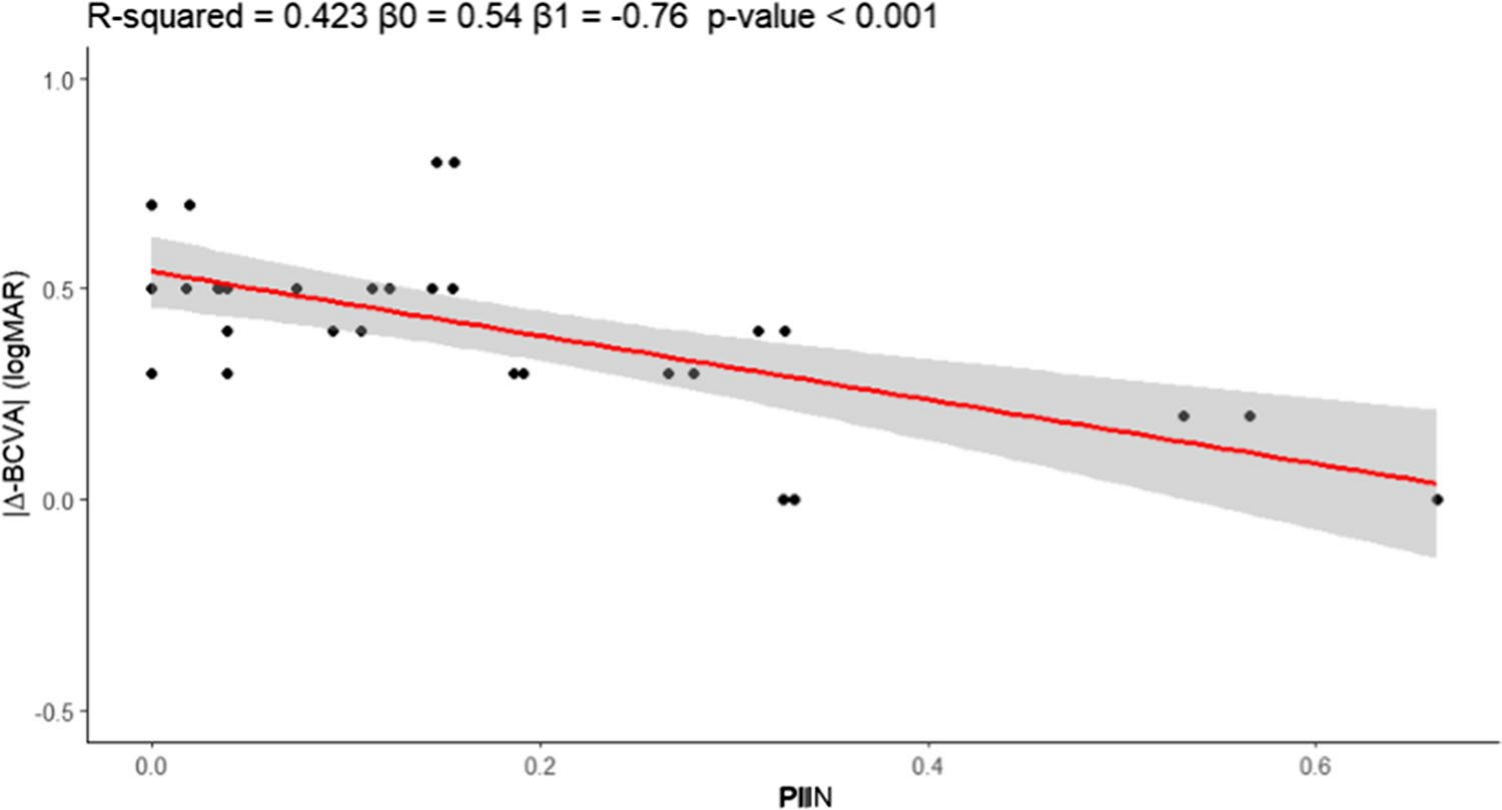
Univariate regression analysis using the ''Photoreceptor Integrity Index'' [PIIN] as a predictor for positive change of BCVA [|∆-BCVA| [logMAR]] over time [follow-up vs baseline]. The performance is expressed by adjusting R2, and the p-value for the regression coefficient is reported

The median lumen hole area was 0.15 mm2 (0.06-0.25), median PRA was 0.10 mm2 (0.06-0.16), and median TA was 0.30 mm2 (0.12-0.42). Median PIIN was 0.17 mm2 (0.05-0.32). Table 1 resumes the patient’s characteristics for en-face parameters. The Spearman ρ correlation coefficient analysis showed that PIIN had a very strong positive correlation with the TA (ρ = 0.95, *p* = 0.001). There was also a strong positive correlation between PIIN and the minimum diameter (ρ = 0.77, *p* = 0.001). Additionally, PIIN was significantly correlated with the baseline ELM (ρ = 0.93, *p* = 0.001) and baseline EZ (ρ = 0.53, *p* = 0.004). The correlation between PIIN and preoperative BCVA was not significant; on the contrary, the higher the PIIN, the higher ∆-BCVA after six months (ρ= -0.58; *p* =0.001) (Figure 4). Finally, there was a significant positive correlation between PIIN and the change in ELM (|∆-ELM|) (ρ = 0.382, *p* = 0.045). Table 2 shows all ρ correlation coefficients between postoperative PIIN and OCT B-scan/en-face parameters.

**Table 2:** Spearman ρ correlation coefficient with listwise deletion to assess the relationship between PIIN and anatomical and functional metrics

	Spearman ρ correlation coefficient	*p*-value
Minimum Diameter (µm)	**0.770**	**0.001**
TA (mm^2^)	**0.948**	**0.001**
BCVA (logMAR) at T0	0.099	0.616
|∆-BCVA| (logMAR)	**−0.578**	**0.001**
EZ (µm) at T0	**0.530**	**0.004**
|∆-EZ| (µm)	0.014	0.945
ELM (µm) at T0	**0.928**	**0.001**
|∆-ELM| (µm)	**0.382**	**0.045**
Months of Symptoms	**−**0.008	0.970
Age, years	**−**0.261	0.180

*TA* Total Area, *PIIN* Photoreceptor Integrity Index, *BCVA* Best Corrected Visual Acuity, *EZ* Ellipsoid Zone, *ELM* External Limiting Membrane. |∆|=change from follow-up (T1) to baseline (T0) in absolute value

### Interobserver agreement

The reproducibility was evaluated on Luminal Area (mm^2^), Photoreceptor Area (mm^2^) and Total Area (mm^2^). The CCC for Luminal Area (mm^2^) was 0.997 (95%CI: 0.994-0.0.998), for PRA (mm^2^) 0.962 (95%CI: 0.929-0.978) and for Total Area (mm^2^) 0.995 (95%CI: 0.989-0.997).

## Discussion

This study used en-face OCT images to characterize photoreceptors observed at the edge of idiopathic MHs. We propose the Photoreceptor Integrity Index (PIIN), an index reflecting the residual preoperative photoreceptor cells on the hole hedge, which could be used to predict postoperative visual outcomes.

As the distance of the MH borders from the RPE increases due to alterations of the vitreoretinal interface, there may be a decrease in the oxygen supply to the photoreceptors and a reduction in the availability of nutrients.

Moreover, compared to the base of the hole, the retina at the MH's edge is likely more prone to increased wall shear stress supported by vitreous dynamics from eye movement [14].

**Fig. 5:**
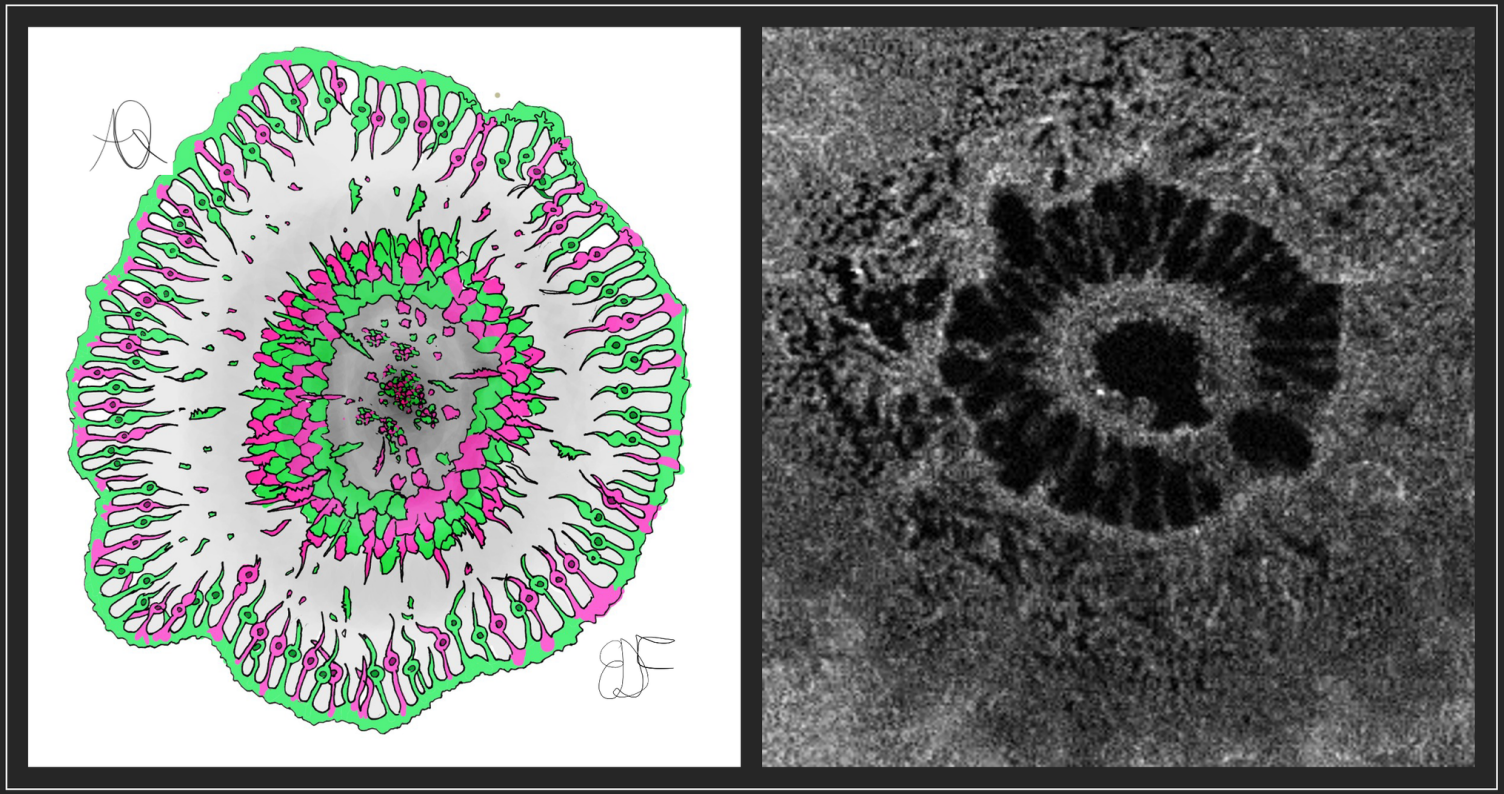
Cartoonized representation of the macular hole from a bird-eye view and comparison with en-face OCT scan show densely packed photoreceptors at the border of the hole, with disrupted ones that fall off the border and lie over the retinal pigmented epithelium [RPE] as remnants. Considering the oxygen’s role in photoreceptors nutrition through choroid, at the maximum height of neurosensory detachment and by focusing at the end of the external limiting membrane, the hypoxic phenomenon may be exacerbated due to the distance from the first oxygen and nutrients source

The PIIN considers the photoreceptors at the highest edge of the hole, i.e. the highest point of ELM, where the oxygen deprivation is more severe [10], as illustrated in Figure 5. The index is obtained by dividing the photoreceptor area calculated by means of the en-face scan by the luminal area of the hole, providing a standardized measure applicable for different MH sizes. To the best of our knowledge, this is the first index described in the literature that considers a cellular component to predict changes in postoperative BCVA. Previous works explored the possibility of considering macular deformation parameters to predict postoperative functional outcomes. The macular hole index proposed by Kusuhara et al. [8] consists of the ratio between hole height and basal diameter. Also, Ruiz-Moreno et al. [2] proposed additional indexes, such as tractional hole index and diameter hole index obtained from B-scan OCT, relying on macular deformation and accounting for tangential and anteroposterior forces in macular hole development. All these indexes were based on MH structural macroscopical characteristics, without considering a cellular component. In addition, OCT B-scan indexes based on linear metrics can estimate tangential and tractional components involved in MH formation but do not consider quantitative photoreceptor disruption and loss. From a pure mathematical standpoint, an index oscillates between 0 and 1 values where the higher the index, the better. Our index is different from the well-known previous indexes since it introduces normalized measures.

Recently, Spaide proposed that MH healing reflects the foveation process, with an outward migration of the inner retinal layer and inward migration of the photoreceptor, with associated improvement in visual acuity over time [15]. This potential could be higher if the residual photoreceptors and retinal layers are more preserved, leading to optimal visual acuity. Beyond foveation theory in restoring visual function, the contracting scar tissue from macular hole closure allows residual packaging of photoreceptors to self-organize to compensate for the lack of effective replication of the macula's highly specialized postmitotic photoreceptor cells. Our study confirms this potential, as the PIIN significantly correlates with postoperative EZd, where the higher the PIIN, the smaller the EZ residual defect will be. A higher PIIN may suggest a higher probability to have a better visual outcome due to higher availability of photoreceptors in external bands reconstruction. It is well known that post-operative EZ correlates with final BCVA. In our study PIIN correlates with both, so the higher the PIIN the higher BCVA recovery from baseline.

The novel index may impact surgical prognosis, starting from the idea that our work demonstrates and quantifies photoreceptor disruption at the borders of FTMHs.

Our work may have some implications in clinical practice. Currently, no parameters quantify the number of photoreceptors lost in the macular hole. In addition, this novel finding may suggest a proper timing for surgery, but probably after a large prospective study that could validate our index.

The novel index might help to understand when the damage to photoreceptors becomes irreversible, confirming Govetto's findings [9].

The present study has several limitations. First, the absence of small MHs makes the index questionable in these cases. Second, data regarding the duration of symptoms could pose challenges when considering the number of cells at the border, as many patients cannot distinguish the exact time of vision drop. Third, the sample size was relatively small, necessitating further validation of the index in a larger population. Metamorphopsia improvements were qualitatively evaluated with the Amsler test, lacking a quantitative test such as M-Chart. However, because the study aimed to demonstrate a photoreceptor loss, this data was considered additional support for a good visual prognosis.

The seven cases that failed to close were excluded from the final analysis because the study's focus was on understanding how photoreceptor integrity, as assessed by the PIIN, relates to successful postoperative outcomes. The PIIN is particularly relevant in cases where the hole has closed, as it is designed to evaluate the contribution of surviving photoreceptors at the hole edge to visual recovery. Further studies with larger samples are needed to understand the PIIN’s role in evaluating surgical failure and its effectiveness across different MH dimensions. Moreover, although any potential interactions between phacoemulsification and functional outcomes in patients with were previously excluded [16], further studies should evaluate possible associations with quantitative measures. In conclusion, the preoperative PIIN appears to be a reliable index representing the preoperative macular photoreceptor at the hole hedge, with a potential for visual outcomes in eyes with full-thickness idiopathic macular holes. Further studies comparing different indexes are warranted to validate their strength.

## Data Availability

Data and results supporting this study’s findings are available upon reasonable request to the corresponding author.
